# Method and application of information sharing throughout the emergency rescue process based on 5G and AR wearable devices

**DOI:** 10.1038/s41598-023-33610-4

**Published:** 2023-04-18

**Authors:** Mengying Wang, Hong Ji, Mo Jia, Zhen Sun, Jinyi Gu, Haiying Ren

**Affiliations:** 1 Information Management and Big Data Center, Peking University Third Hospital, Beijing, China; 2 China Academy of Information and Communications Technology, Beijing, China

**Keywords:** Engineering, Electrical and electronic engineering

## Abstract

The 2022 Winter Olympics were held in the three competition zones of Beijing, Yanqing and Zhangjiakou, China. The venues of this Winter Olympics were scattered and the terrain was complex. Moreover, the medical resources of Hebei and Beijing were relatively unbalanced. In the medical security of major events, the connection between first aid and in-hospital processes is of the utmost importance to rescue quality. 5th generation mobile network (5G) applications in medical scenarios are on the rise. It would be of great relevance to fully use 5G’s low-latency and high-speed features to share the process information of patients, ambulance personnel, and the destination hospital’s rescue team at emergency scenes and in transportation, improving rescue efficiency. This paper proposes a system scheme of cross-institutional emergency health information sharing based on 5G and augmented reality wearable devices. It also integrates the construction method of monitoring and other sign data sharing, in addition to testing the proposed scheme’s service quality in 5G environments. In the deployment area of the 5G emergency medical rescue information sharing scheme for the Beijing Winter Olympic Games, we selected two designated medical support institutions for testing. The test adopted a combination of fixed-point and driving tests to experiment on the service data, voice service, and streaming media indicators. The 5G signal's coverage rate was close to 100%, the standalone connection's success rate was 100%, and the drop rate was 0. The average downlink rate of multiple scenarios was 620mbps, and the average uplink rate of 5G was over 71.8mbps, which is higher than the average 5G level in China. The downlink rate was more than 20 times larger than the 4th generation mobile network (4G) rate. This study’s proposed scheme demonstrates the importance of 5G applications in emergency response and support, in addition to providing a suitable scheme for the integration of 5G networks in the medical scene.

## Introduction

The 2022 Winter Olympics were held in China across three zones: Beijing, Yanqing, and Zhangjiakou. The event’s ice and snow sports were characterized by strength skills, fast speed, and intense confrontation, making players prone to sports injuries and possibly high-violent trauma^1,2^. The venues of this winter Olympic Games were scattered and the terrain was complex, while the medical resources in Hebei and Beijing were relatively unbalanced. The level of medical security in Yanqing and Zhangjiakou competition areas was arguably inadequate, leading to an objective need of transporting injured players to high-level medical institutions for treatment. Therefore, in this winter Olympic Games, the task of medical emergency support is arduous, and patients are bound to be transported from the competition area hospital to the guaranteed hospital for treatment. In the medical security of major events, the poor connection between pre-hospital and in-hospital emergencies is one of the most important factors reducing treatment quality^3^. In the emergency process, through the establishment of information sharing channels between the stadium, ambulance, designated hospital, and transportation hospital, experts from multiple institutions can quickly participate in the whole pre-hospital emergency process, recognize any real-time changes in the vital sign information of the injured, and intervene in advance to guide the emergency doctors to deal with the special treatment. This is particularly important for dealing with major emergency public health events having the significance of special guidance^4,5^.

Presently, the scene’s working mode including the ambulances and hospitals in China's emergency system is a single-threaded serial rescue. In the traditional pre-hospital emergency process, the emergency command center receives the call for help and inquiries about the patient's condition. According to the location, injury type, symptom level, and previous data, the ambulance is deployed to transport the patient to the designated medical institution. However, in the emergency support of sports competitions, patients are usually transported and treated between designated emergency institutions^6^, and there is a lack of research on transportation information sharing methods between them. In the process of emergency transportation, the treatment mainly depends on pre-hospital doctors^7,8^. The main emergency information sharing is still communicated with the in-hospital reception experts through telephone or vehicle-mounted cameras. The patient’s condition and physical signs monitoring need to be manually entered into the medical record system. Judging the injury in pre-hospital emergencies depends on the personal experience of pre-hospital doctors. Due to the latter’s variances in personal abilities and medical levels, there will be some cases where the injury’s focus is not prominent or where important symptoms are missed. Moreover, there will be some others where the ability to deal with emergencies is insufficient, resulting in injury miscalculations and potentially incalculable consequences^9^. At present, the pre-hospital emergency process is hindered by inadequate communication conditions and information levels. Additionally, there is a lack of effective, accurate, and timely means of delivery to designated hospitals. As a result, the in-hospital emergency experts can only infer the changes in the patient's injury by phone calls from the pre-hospital doctors without directly judging injuries through objective examinations, such as electrocardiogram monitoring and ultrasound to give specific opinions. Additionally, the in-hospital treatment is relatively passive.

To improve information transmission in the emergency process, previous studies have applied wireless communication technologies to the ambulance service^10^. Rehman et al.^11^ suggested that cellular base stations and transmitters should be deployed during transportation to transmit the ultrasound conditions in ambulances to the receiving hospital in video streams. Mukhopadhyay et al.^12^ developed a medical information transmission system based on a wireless communication system to transmit the patient’s vital sign data to the receiving hospital; the system has been tested under the 4G network. Cao et al.^13^ deployed on-board cameras on ambulances, but had the following shortcomings: it was impossible to locate and view the patient’s detailed condition changes, such as skin color changes and wound details. Zhai et al.^14^ built an emergency transport system with the assistance of 5G, but the information sharing only included audio and video and lacked the cross-medical institution sharing of the patient’s medical records and sign monitoring data. Moreover, it was only tested in the laboratory and not in real scenarios. Therefore, emergency transportation using traditional wireless communication technologies still faces some challenges. First of all, the ambulance’s remote guidance requires real-time video and audio communication, and the Quality of Service (QoS) and Quality of Experience (QoE) of ordinary 4G networks cannot cope with stable and continuous remote guidance^15^. Second, medical data involves patients’ important personal privacy. From the perspective of personal information protection, medical data need be transmitted only via private network environments to reduce the risk of leakage associated with transmission via the Internet. Third, in terms of the emergency process and information sharing, the emergency scene will transmit video and audio to the emergency commander and the receiving doctor for the first time. The electrocardiogram monitoring data monitored by the ambulance in real-time during the transportation should be transmitted to the receiving doctor in real-time to intervene in the rescue in advance.

5G is a new generation of cellular mobile communication technology with a high rate, wide spectrum, low latency, internet of things, mobile edge computing, network slicing, and high-precision positioning. The use of smart antennas in 5G enables the transmission of signals in the direction of the user's location, maximizing signal strength and signal-to-noise ratio and achieving the most effective space-division multiple access (SDMA) process for wireless signal transmission and reception^16^. The transmission rate of 5G is 10–100 times higher than that of 4G. The peak theoretical transmission rate can reach 10Gps, and the time delay is 9/10 lower than that of 4G^17,18^. The comparisons of key performance indicators between 5 and 4G are presented in Table 1. In previous studies, 5G has been mainly used in online counseling, online health monitoring, remote diagnosis, and mobile robotic surgery^19–21^. Several studies^22,23^ have provided solutions to improve the security of medical systems, some of which focus on multiple heterogeneous network settings^24^. 5G also has immense potential in machine-to-machine communication and interconnection. For example, 5G can connect a large number of IoT devices and robots, providing better control and response speed for industrial equipment and robots, enabling online real-time communication and collaborative work, and thus improving production efficiency and flexibility in multiple industries^25,26^. In summary, it could significantly solve the problem of communication efficiency in the process of mobile emergency transportation and ensure security. Utilizing technologies such as Mobile Edge Computing (MEC) and network slicing, 5G medical customized networks can design dedicated access for hospital and inter-hospital services, and are compatible with virtual private network (VPN), Internet, cellular, and satellite. However, in addition to high-bandwidth and low-latency communication methods, the emergency process also needs to be efficiently reconstructed through information technology. To make better decisions before the patient’s admittance to the hospital, we could interact with his/her situation at the real-time emergency scene, share heterogeneous information, such as the geographic location, patient medical records, medical examinations in emergency transportation, and use positioning technology and 5G to help referral hospital experts obtain the information transmitted by the pre-hospital first-aid location, real-time video, medical status, etc.

**Table 1:** Comparisons of key performance indicators between 5 and 4G.

Key performance indicators	Definition	4G	5G
User experience rate (bps)	The lowest transmission rate available to users in the real network environment	10 Mbps	100–1000 Mbps
Connection density (/Km^2^)	Total number of online devices supported per unit area	100,000/km^2^	1,000,000/km^2^
End-to-end delay (ms)	The correct receiving time of the data packet from the source node to the destination node	10 ms	1 ms
Mobility (Km/h)	Maximum relative movement speed between the transmitter and receiver when certain performance requirements are met	350 km/h	500 km/h
Flow density (bps/Km^2^)	Total flow per unit area	0.1 Mbps/km^2^	10,000 Mbps/km^2^

The main contribution of this study is proposing a whole-process emergency solution to support 5G and test its performance in practical application scenarios. (1) A plan for sharing emergency information among multiple medical institutions using 5G and BeiDou Navigation Satellite^27,28^ is proposed, reflecting the value of 5G in emergency response and security. (2) A rapid sharing application scenario of emergency field information based on 5G and AR smart wearable devices is proposed, freeing doctors and improving pre-hospital emergency communication efficiency. (3) In the real application scenario, the 5G performance was verified through the Pioneer software and the GSP dotting collector to test the service indicators, voice service indicators, and streaming media services. Moreover, the superiority of our proposed command and emergency full-process information sharing scheme was verified.

## The 5G system architecture for smart emergencies

### The smart emergency’s overall system designing

**Figure 1:**
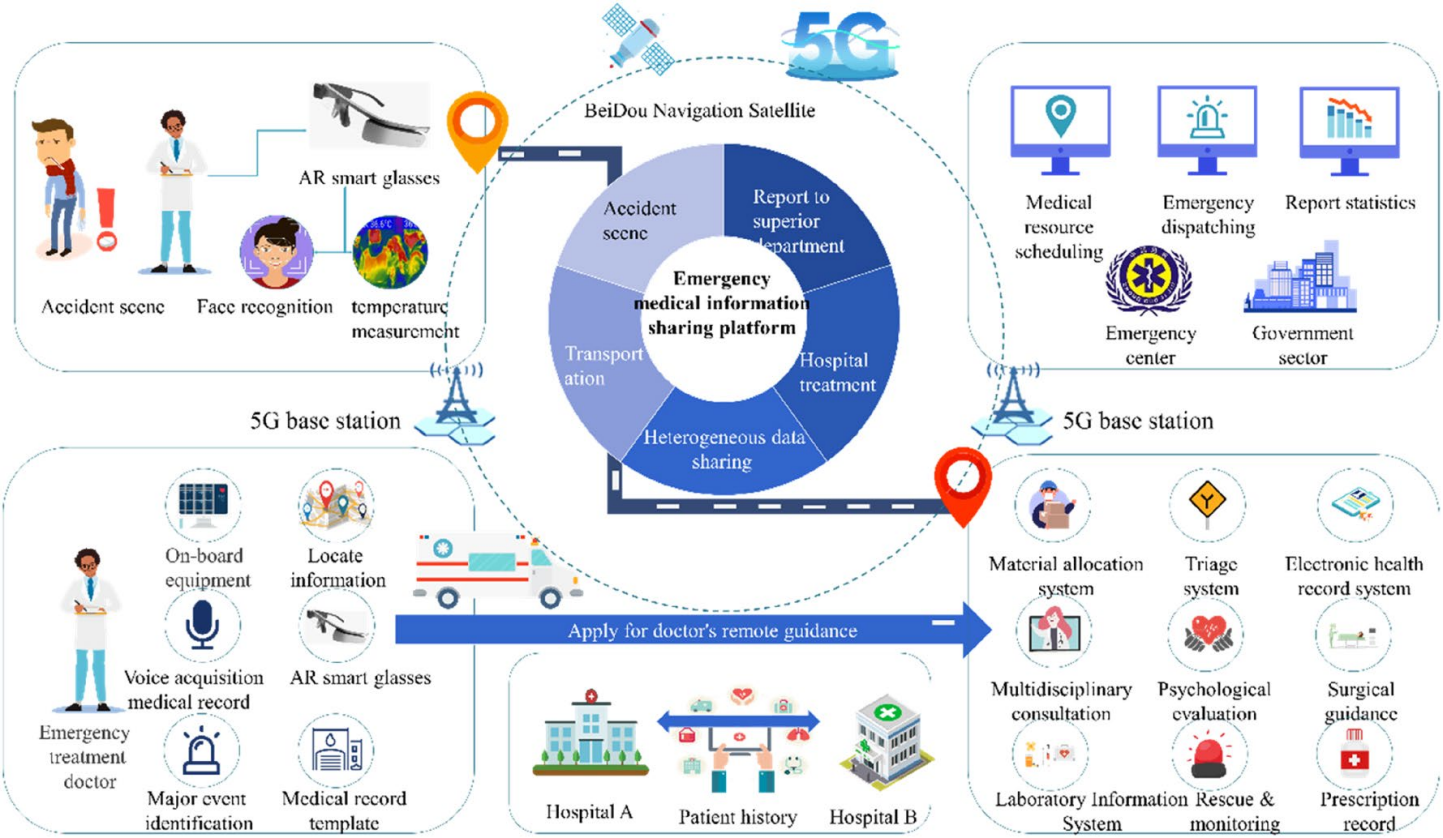
The 5G-enabled smart ambulance proposed architecture.

**Figure 2:**
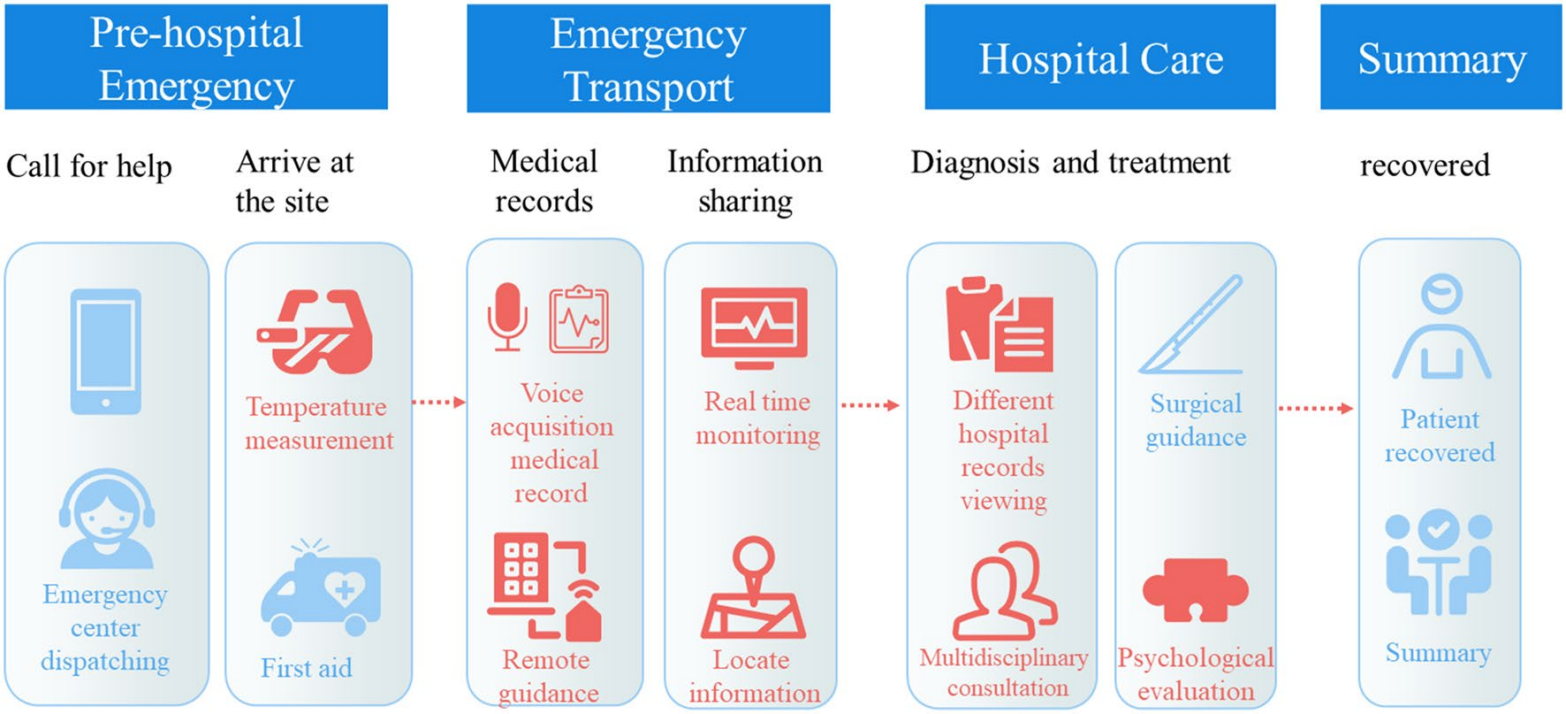
The emergency treatment flow chart (the red part is new in this study, and the blue part is the general emergency flow).

Based on the experience of previous international large events, the particularity of emergency medical rescue, and the overall medical security principle of the Winter Olympics^1,29^, we can summarize this study’s proposed smart emergency information sharing scheme as ‘1234N”. Its structure is shown in Fig. 1. It is composed of one platform (the emergency medical information sharing platform), two basic implementations (5G and BeiDou), three terminals (the AR smart glasses, vehicle tablet, and computer workstation), four emergency process scenarios, and N extended interactive applications covering the complete emergency process from the first-aid scene, transportation process, hospital treatment, and post-hospital summary. The emergency process is shown in Fig. 2. We designed the platform to increase the rapid identification function of major emergencies, such as biological and chemical poisoning events, to support major events in the Winter Olympic Games.

This study’s application covers two medical institutions, including the Third Hospital of Peking University in Beijing and the hospital of Yanqing District. They were the designated support hospitals for the Winter Olympics and undertook the tasks of resident medical stations, emergency transportation, hospital reception, and treatment during the Winter Olympics. The electronic health information systems in them are heterogeneous and need to interact through the medical information sharing platform proposed in this study.

In this study, we built the 5G medical private network for the emergency rescue support of the Winter Olympics. The construction methods include physical and virtual private networks. During the construction of the prior, the base station and core network elements were sunk into the hospital to realize its internal network isolation. During the construction of the latter, MEC and network slicing were deployed to sink the User Port Function (UPF) to the hospital’s vicinity. The 5G private network’s overall architecture can be divided into three levels: the wireless, bearer, and core networks. The 5G medical private network carries the diversified business needs such as vehicle positioning, audio, and video interaction, medical data information sharing, medical resource scheduling, and remote treatment guidance in the process of ambulance service, and provides reliable network support for the in-depth integration of data, resources, and services.

#### The 5G network slice

**Figure 3:**
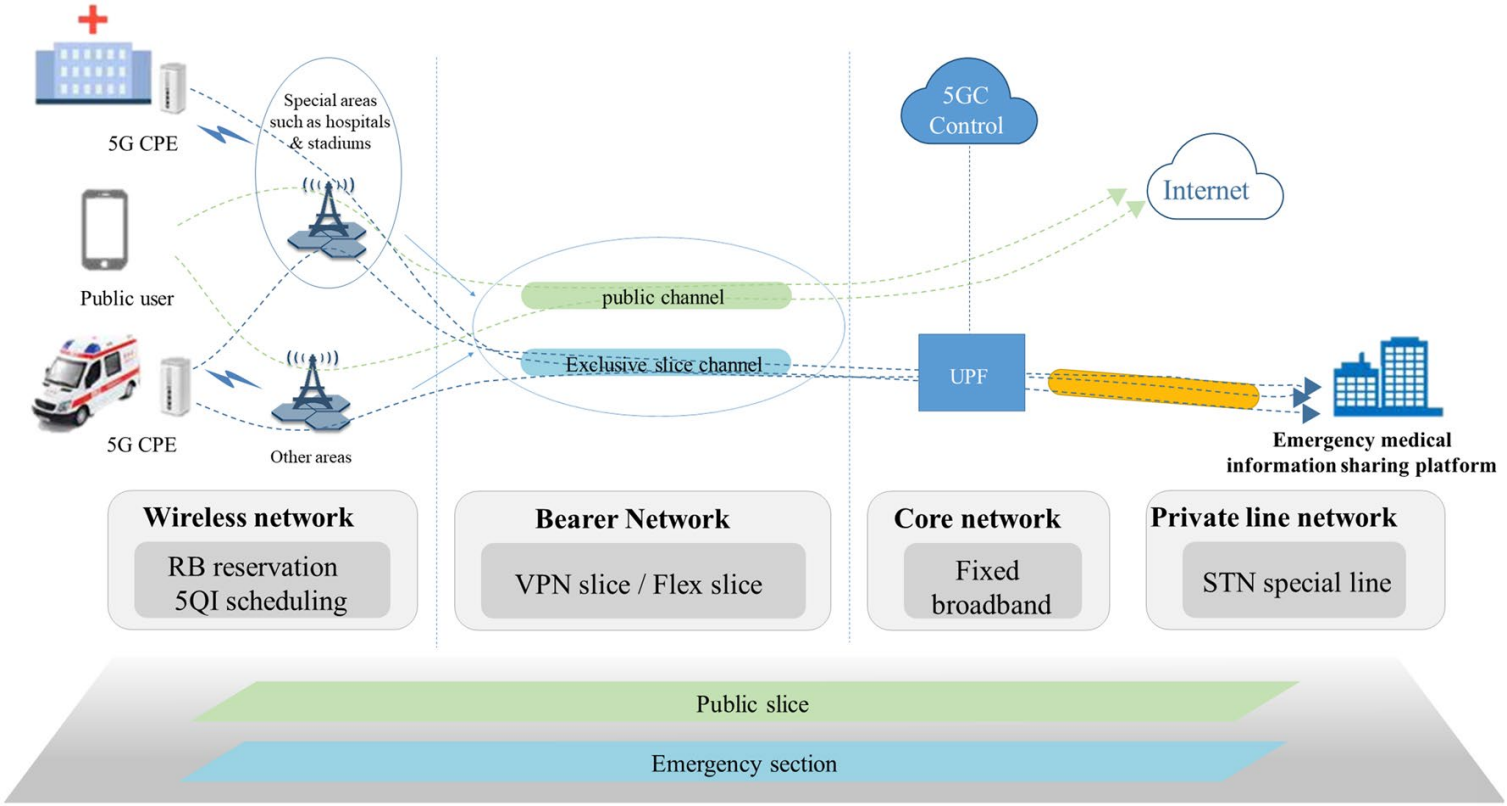
The 5G slice dedicated line networking architecture.

According to different business scenarios of 5G pre-hospital intelligent first aid, network slices can be customized. Soft or hard slices can be deployed in sections according to business needs. The slice networking architecture is shown in Fig. 3.

Wireless network sub-network slicing: the wireless side configures dedicated slices for emergency services at all access base stations. Since the 5G pre-hospital emergency scene involves special fields and other public areas where emergency services are concentrated, such as emergency hospitals and Olympic venues, and other public areas where ambulances pass through, the wireless network can provide slice-level security isolation and wireless resource protection using Resource Block (RB) reservation Technology. At the same time, the differentiated QoS scheduling technology is combined to guarantee the refined service.

Bearer network subnet slicing: the bearer network uses VPN soft slicing/flex to establish a dedicated slicing channel for emergency services, providing higher security isolation and bandwidth assurance services.

Core network subnet slicing: since the emergency service is a wide-area access one, the core network side uses the provincial center’s shared network element to conduct the service. It configures emergency service dedicated slices and customized Data Network Name (DNN) on the core network’s shared network element, in addition to configuring the fixed bandwidth on the N6 port to realize service isolation and resource guarantee^30^.

The fixed mobile convergence special line binds the Smart Transport Network (STN) at the core network’s exit to establish a dedicated transmission link between it and the emergency platform to realize safe and reliable transmission from the 5G terminal to the platform.

#### Resource block reservation

**Figure 4:**
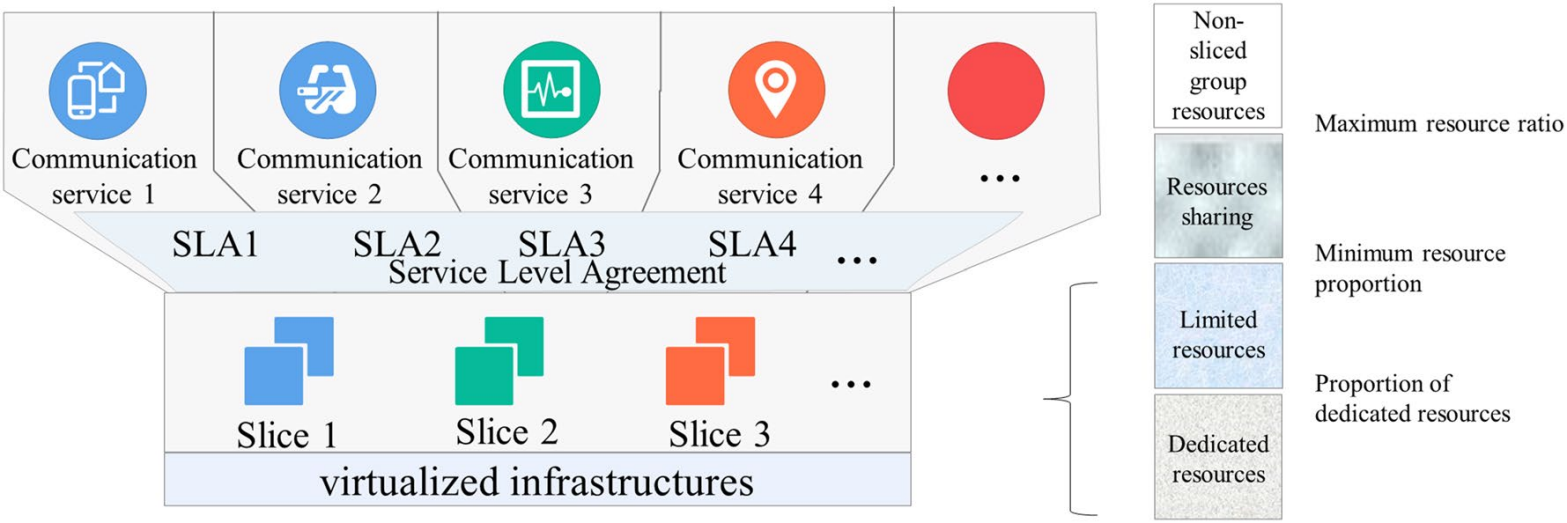
A schematic diagram of the 5 g slice group resource reservation.

The RB resource reservation technology allows multiple slices to share RB resources in the same area and allocate varying physical resource block shares to different slice groups. The base station scheduler schedules resources based on the allocated shares to ensure that one slice group’s resource shortage does not affect another’s service quality, achieving a certain degree of resource isolation. The 5G slice group’s RB resource reservation diagram is shown in Fig. 4. The 5G pre-hospital emergency scene mainly involves the emergency zone, the Olympic Stadium, other business-concentrated public areas, and other public areas where ambulances pass. Special areas of business concentration include Peking University Third Hospital (the expert group provides remote guidance, teaching, and consultation) and Yanqing Hospital (resident treatment and transportation hospital). For special areas where services are concentrated, we can reconfigure the RB resource reservation for dedicated slices of emergency services on the field service base station to improve service transmission resource guaranteeing.

### Heterogeneous data sharing scheme of hospitals

**Figure 5:**
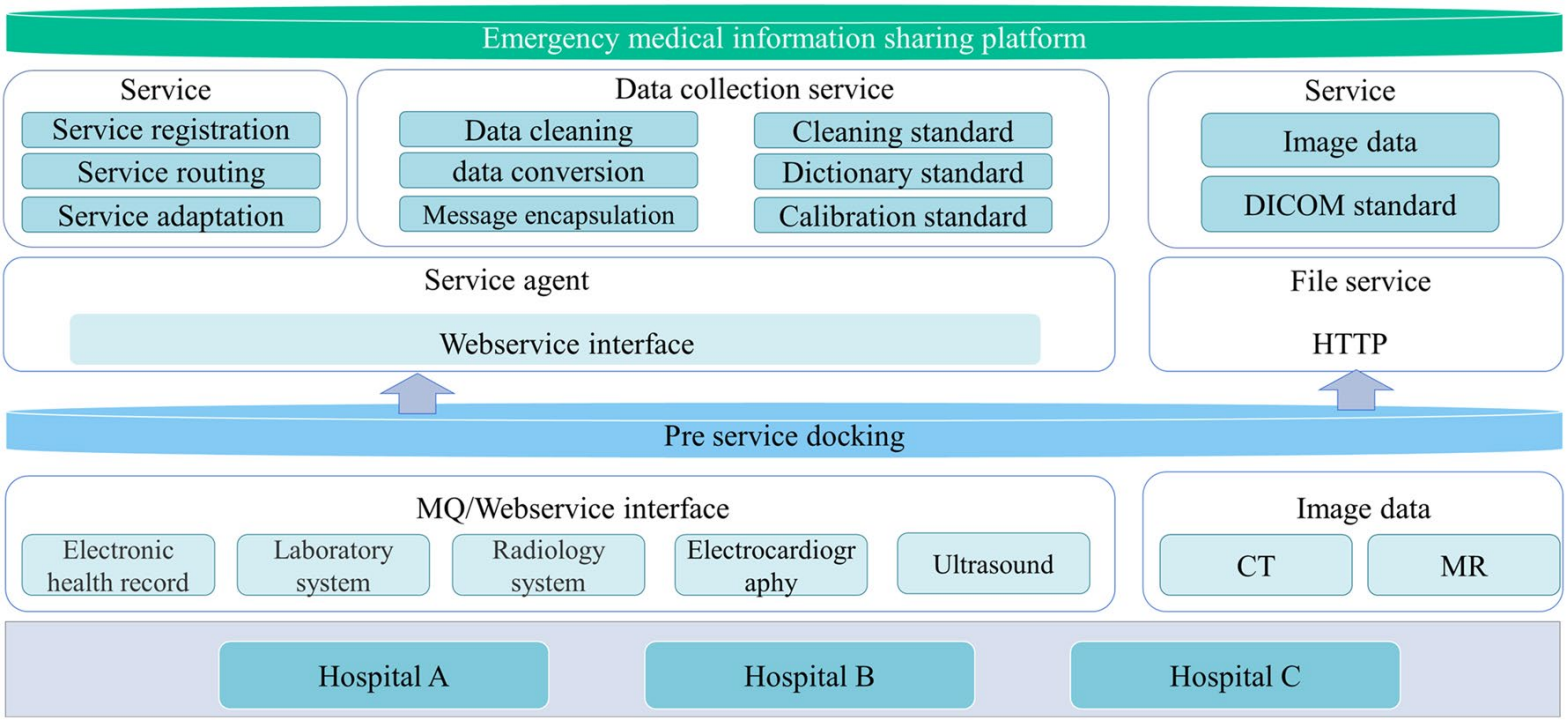
The front server-based medical information sharing platform’s interactive architecture.

In the process of emergency transport and in-hospital treatment, over 50% of doctors believe that the patient's medical history is the most decisive aspect of rapid diagnosis^31^, leading to the need for cross-institutional medical record information sharing. Heterogeneous medical data have different data structures and different sources caused by heterogeneous electronic medical record systems^32,33^. In this study, we adopted the pre-service docking scheme to complete the information exchange between the medical information sharing platform and various medical institutions. Medical institutions organize data according to this platform’s information exchange requirements. After encapsulating data with the extensible markup language (XML) data of the Health Level Seven (HL7) standard, they interact with the medical information sharing platform via message queuing. The interaction data is shown in Table 2. In the process of front server interaction, the medical information sharing platform provides standard interactive service interfaces, such as restful, json, etc. Medical institutions then convert the organized data into Message Queue (MQ)^34,35^ by calling the standard interfaces for interaction. Figure 5 shows the interaction scheme.

**Table 2:** The shared data between the different hospitals.

Category	Specific fields							
Prescription	Identity ID	Drug code	Route of administration	Physician signature	Start date	Use frequency	Drug name	Drug specifications
Blood Transfusion	Identity ID	Blood group	Transfusion timing	Transfusion components	Transfusion volume	Transfusion reactions		
Monitoring Data	Identity ID	Monitoring items	Monitoring time	Monitoring outcomes				
Anesthesia Record	Identity ID	Anesthesia modality	Anesthesia time	Anesthesia records				
Surgical Record	Identity ID	Surgery code	Name of surgery	Operative time	Operative notes			
Treatment Record	Identity ID	Treatment items	Treatment code	Treatment time				
Treatment Request	Identity ID	Treatment items	Treatment code	Request time				
Image	Identity ID	Examination site	Inspection images	Inspection time	Outpatient/inpatient			
Inspection Report	Identity ID	Examination site	Inspection items	Inspection item code	Diagnosis	International Classification of diseases-10	Outpatient/inpatient	Report time
Inspection Request	Identity ID	Examination site	Inspection items	Inspection item code	Diagnosis	international Classification of diseases-10	Outpatient/inpatient	Request time
Health Record	Identity ID	Record title	Contents	Record time	Departments	Outpatient/inpatient	Physician signature	
Test Request	Identity ID	Item code	Specimen type	Request time	Outpatient/inpatient			
Test Report	Identity ID	Name of the test item	Item code	Results reference values	Specimen type	Test results	Report time	Outpatient/inpatient
Nursing Assessment	Identity ID	Assessment item	Item code	Assessment result	Assessment time			
Vital Signs	Identity ID	Signs items	Item code	Sign values	Vital signs measurement time			
Dictionary of Medicines	Drug name	Drug code						
Diagnostic Dictionary	Disease Diagnosis	international Classification of diseases-10

### The 5G intelligent ambulance’s reconstruction

**Figure 6:**
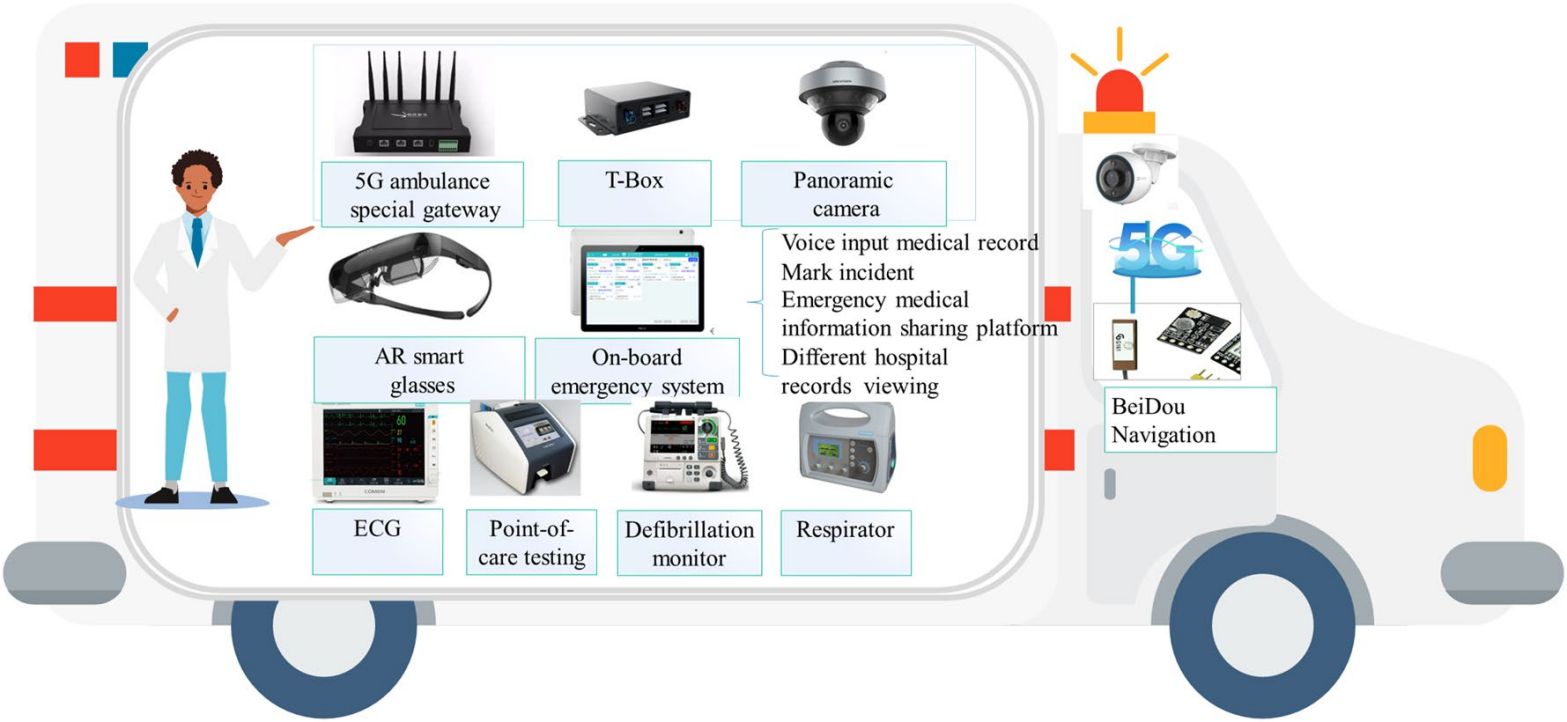
A schematic diagram of the reconstruction and deployment of ambulances.

The reconstruction of the 5G ambulance is based on the 5G network. It mainly involves the 5G ambulance’s special gateway (access to medical equipment), T-box equipment (the intercommunication equipment between the vehicle and cloud platform), BeiDou positioning equipment, ambulance information terminal (the medical record system on the pad), emergency medical equipment, and other on-board equipment. As shown in Fig. 6, the 5G network connects all equipment to realize centralized management and information sharing. The 5G ambulance-mounted special gateway is key equipment for vehicle information upgrading. It carries the ability of data transmission between 5G wireless communication data transmission and various on-board devices with different communication interfaces and data formats. The 5G ambulance-mounted special gateway provides large memory, supports 4 K video smooth playback, software operation, and calculation, can provide end-to-side edge processing with the edge cloud, and uniformly accesses various devices in the ambulance, such as a vehicle-mounted tablet and an electrocardiograph. We designed the T-box information collection box on the Linux kernel. The box transmits data of on-board medical equipment to the server side of the information sharing platform to connect the data of different medical examination and monitoring equipment via intelligent interconnection.

### The 5G-based intelligent application of the emergency process

#### Accident scene emergency fast identification of 5G and AR smart glasses

**Figure 7:**
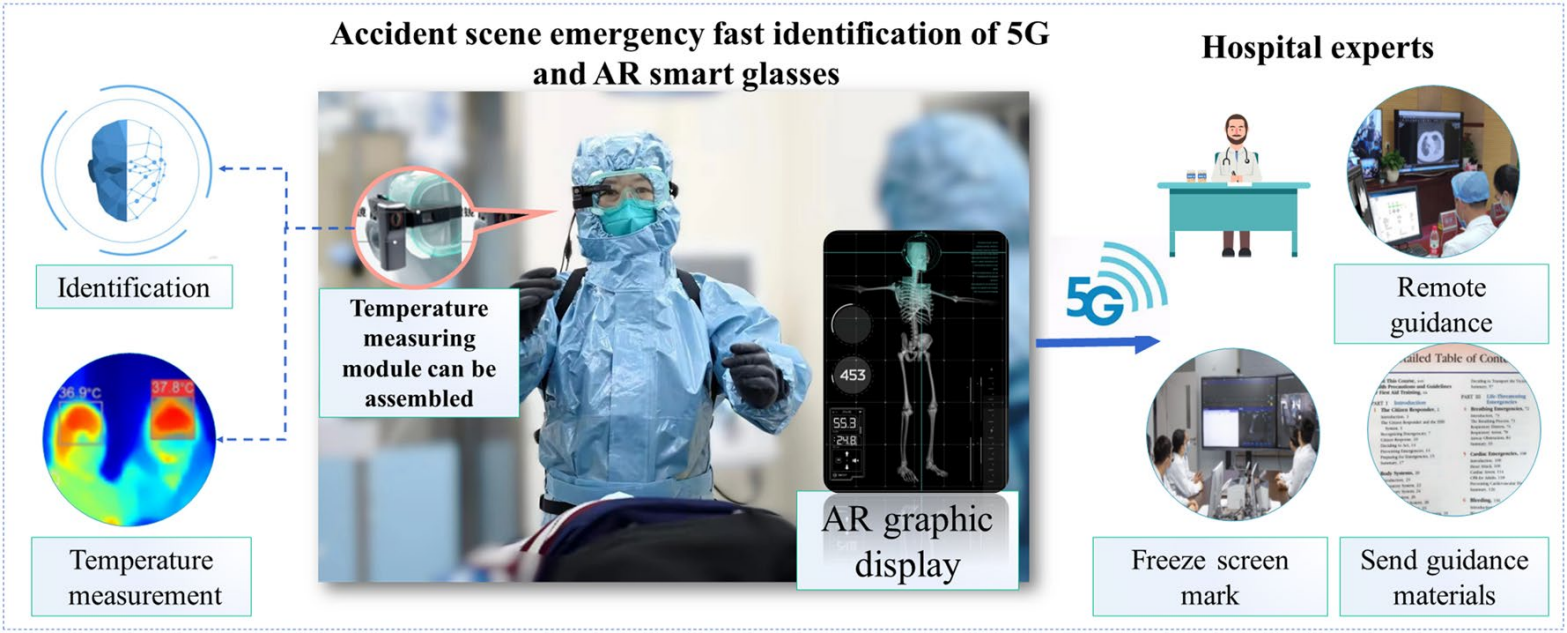
AR smart glasses applications.

Recent studies have shown that AR has great potential in medical scenarios^36,37^. It can directly display patient health-related information and view relevant information while paying attention to patients. In study^38^, AR devices were used to classify patients in disaster events, revealing the limitations of Wi-Fi connectivity. In this study, 5G and AR smart glasses were used in the emergency scene, and face recognition technology was applied to quickly identify the information of the injured and the patient's temperature. The patient’s information was then transmitted to the medical information sharing platform in real-time. Rapidly identifying patient identities is particularly important in major events or complex emergency scenes. The face images of key groups of people are collected in advance, which helps identify important groups of people. For example, by combining accessible and contactless body temperature measurements, it is possible during the COVID19 pandemic to quickly pre-check and classify febrile and non-febrile patients. The applications of AR smart glasses are shown in Fig. 7.

#### Intelligent information sharing and remote guidance during transportation 

Location information sharing: the terminal mounted on the ambulance collects satellite positioning information through the BeiDou module to obtain real-time and high-precision emergency vehicle’s positioning longitude and latitude coordinate information while simultaneously mapping its travel route through the API interface of the Gaode map^39^.

Medical record information sharing: the system in this process selects the corresponding task card through the first aid system on the vehicle-mounted tablet while recording the task time axis and other important time nodes, such as the time of receiving the task, the time of on-site arrival, the time of departure, and the time of arriving at the receiving hospital. According to the patient’s condition, the medical record is entered into the emergency system by voice. The system can automatically recognize and convert the voice content into the medical record text and mark the patient’s condition as critical, severe, or mild. This facilitates the emergency process, allowing the dispatch center and receiving medical institutions to track and monitor patients with special needs. On the ambulance, the real-time electrocardiogram waveform, ventilator waveform, heart rate, respiratory rate, blood oxygen saturation, pulse, blood pressure, body temperature, and other vital signs are collected and transmitted to the medical information sharing platform through the T-box and 5G network in real-time. The receiving hospital checks the real-time monitoring results in advance and guides ambulance doctors to pay attention to the patient’s condition changes.

Remote guidance: the emergency medical doctor initiates a remote assistance application through AR glasses. Real-time 5G signals are used to transmit the first visual field picture to the receiving expert team. The latter receives the diagnosis online via the workstation and provides real-time online audio-visual guidance. For details of the patient’s wound, the screen can be remotely frozen for AR marking, highlighting key areas of concern. When major emergencies occur and special operations are required for patients, the receiving experts will send important reference information, such as pictures, and the emergency doctors would directly view the operation guide through AR smart glasses. Accordingly, the proposed system not only provides professional graphic visual and intuitive guidance but also remote experts intervention, admitting patients to the hospital as soon as they arrive on the train.

#### Multidisciplinary expert consultation in hospital treatment

To form a consultation report, we integrate the patient's Cross-institutional medical records and image data and conduct online discussions of difficult cases via the multi-disciplinary expert consultation system. During major events, we also consider early assessment and mental health intervention, in addition to the timely treatment of a patient’s physical health^40^. We conduct initial assessment through the risk factor assessment scale of dangerous behaviors, anxiety and depression scale, and intensive care delirium screening form. When the early warning score is exceeded, a psychologist is contacted for early intervention.

## Field test results

### The field test scenario’s overall design

**Figure 8:**
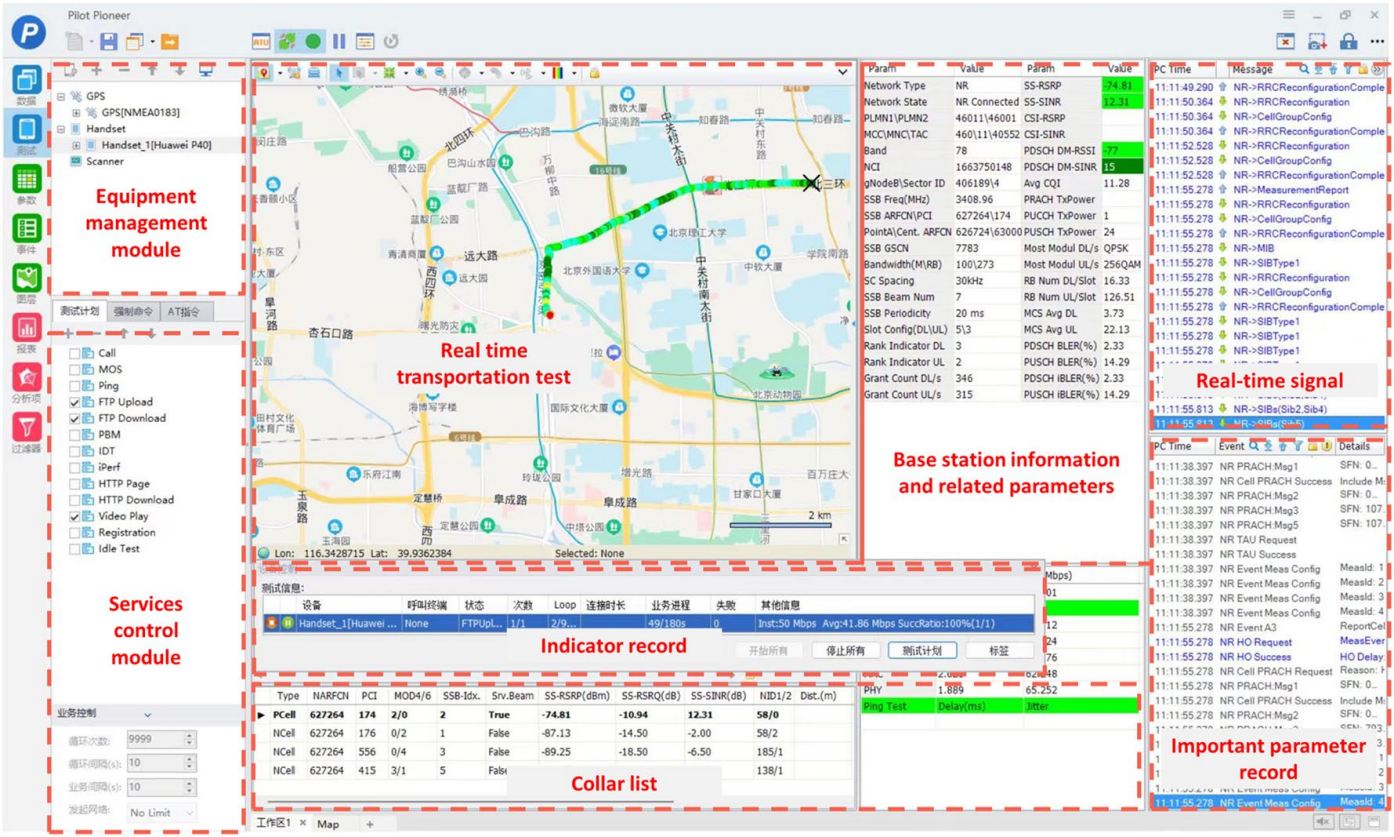
The pioneer software’s interface diagram.

The test adopted a combination of fixed-point and driving tests to test the service data, voice service, and streaming media indicators. We built the test system based on special equipment and software and divided it into two parts: the front-end data acquisition and back-end data analysis systems (Pioneer software). Figure 8 shows the Pioneer software’s interface. According to different test purposes, the front-end data collection system selected the static/walking/vehicle mode, simulated the behavior in emergency scenes, conducted data interaction or continuous dialing, and collected network data. The latter collected data through statistics and analysis of back-end processing software, evaluated the network’s coverage and service qualities, and analyzed its problems.

Test steps: (1) we first connected the test system and terminal. (2) For the testing system’s parameter configuration, we set the download time to 180 s and the upload time to 180 s. For the thread settings, we set 20 threads for downloading and 10 threads for uploading. As for the testing method, we used the cycle-by-time approach. We set the cycle interval to 15 s. For the test times, we selected 1 time for the fixed-point test and an infinite cycle for the driving test. (3) We then conducted file transfer protocol downloading and uploading under the current 5G network environment. For downloading, we used 50 GB dicom image files; for uploading, we did not set the file size. (4) We recorded the test results of all indicators.

The test first-aid scenarios include the first-aid scene, AR smart emergency rescue guidance, transportation guidance, and arrival at the hospital. When selecting the test location, the doctor's office, classroom, and conference room, we considered all clinical departments involved in the multiple potential traumas of the Winter Olympics, such as orthopedics, sports medicine, the emergency department, and operating room. For testing, all five locations were selected and the average value was chosen. In the real application scenario, the 5G performance was verified by the test data indicators. For the definitions of all indicators, check Appendix 1.

### Test results

At the test point, the system and equipment can access the 5G network, carry out various services, and maintain connectivity with other points. In the test area, the Intra-hospital test scenario indicators were satisfactory and the test results are presented in Table 3. The 5G signal’s coverage rate was close to 100%, the success rate of the SA connection was 100%, and the drop rate was 0. The average downlink rate of multiple scenarios was 620mbps, and the average uplink rate of 5G was above 71.8mbps. Except for the operating room, the connection rate of voice service indicators was 100%, the delay and bit error rates were low, and the packet loss rate was almost zero. The evolved packet system (EPS) fallback’s fast return delay was less than 3 s. Moreover, the streaming media indicator data was excellent and the results are listed in Table 4. The performance of media indicator data was excellent. The drop, playback failure, and jam rates were all 0. The maximum delay was 0.03 s and the results are presented in Table 5. The ambulance’s lower downlink speed during transportation was mainly due to problems such as the channel switch and Doppler shift^41^ during its movement, resulting in an unstable data rate.

**Table 3:** Intra-hospital scenario indicator test results.

Category	Indicators	Unit	Emergency Site	Emergency transport	Multidisciplinary consultation Application	Consultation access	Different Hospital records viewing	Application for remote ward rounds	Application for tele-surgery guidance	Tele-surgery Guidance access	Remote hand over to the next shift	Remote education	Summary
Intra-hospital test scenario indicators	Signal coverage	%	100	91.52	100	100	100	100	100	99.86	100	100	100
	SA Connection Success Rate	%	100	100	100	100	100	100	100	100	100	100	100
	SA dropout rate	%	0	0	0	0	0	0	0	0	0	0	0
	SA switch success rate	%	100	100	100	100	100	100	100	100	100	100	100
	5G length of stay ratio	%	100	100	100	100	100	100	100	100	100	100	100
	SS RSRP	dB	− 70.25	− 79.13	− 76.44	− 65.91	− 61.47	− 67.07	− 80.41	− 83.85	− 78.39	− 50.7	− 60.07
	SS SINR	dB	47.824	24.138	62.66	47.46	52.276	64.94	61.56	20.846	49.756	48.566	39.474
	5G downlink average rate	Mbps	801.36	192.63	649.73	693.59	745.91	742.77	568.73	431.99	685.9	700.2	611.26
	5G uplink average rate	Mbps	78.456	72.252	71.5	83.37	60.704	60.52	64.08	60.368	86.226	81.41	71.172
	Packet loss	%	0	0	0	0	0	0	0	0	0	0	0
	Error rate	%	8.94	9.58	9.07	9.06	9.01	8.97	9.03	9.14	8.99	9	8.88

**Table 4:** Voice service indicator test results.

Category	Indicators	Unit	Emergency Site	Emergency transport	Multidisciplinary consultation application	Consultation access	Different hospital records viewing	Application For remote ward rounds	Application for Tele-Surgery Guidance	Tele-Surgery Guidance Access	Remote Hand Over To The Next Shift	Remote education	Summary
Voice service indicators	EPS fallback on rate	%	100	100	100	100	100	100	80	100	100	100	100
	EPS fallback call delay	s	3.46	4.3	6.96	4.54	3.9	4.76	4.65	3.59	5.04	3.98	3.98
	After the EPS fallback hangs up, the fast return delay is less than 3S	%	100	100	100	100	100	100	100	100	100	100	100
	Error rate	%	0.32	1.93	2.17	1.03	0.66	13.16	2.17	2.41	1.47	1.44	1.35
	RTP packet loss	%	0	0	0.02	0	0	0	0	0	0	0	0
	RTP end-to-end delay	s	0.03	0.03	0.08	0.02	0.04	0.02	0.04	0.03	0.02	0.05	0.04

**Table 5:** Streaming media service indicator test results.

Category	Indicators	Unit	Emergency Site	Emergency transport	Multidisciplinary consultation application	Consultation access	Different hospital records viewing	Application for remote ward rounds	Application for tele-surgery guidance	Tele-surgery guidance access	Remote hand over to the next shift	Remote education	Summary
Streaming media service indicators	Drop rate	%	0	0	0	0	0	0	0	0	0	0	0
	Playback start failure rate	%	0	0	0	0	0	0	0	0	0	0	0
	Play stuck rate	%	0	0	0	0	0	0	0	0	0	0	0
	Play start delay	s	0.02	0.02	0.015	0.031	0.031	0.016	0.015	0.046	0	0.021	0.012
	RTT delay	s	150	150	150	150	150	150	150	150	150	150	150
	Video Play vMOS	/	2.15	2.15	2.15	2.16	2.16	2.15	2.16	2.15	2.16	2.16	2.16

## Discussions

### The actual scenario test results satisfy the needs of innovative medical applications

This study has fully tested the real scene indicators. Results demonstrate that this study’s proposed scheme can stably provide downlink and uplink data rates of around 620 and 72 Mbps, respectively. In the specific voice and streaming media services, the packet loss and bit error rates were almost zero. The 5G medical network can support almost all innovative medical applications defined in next-generation mobile networks^42^. Thus far, based on actual applications, the simultaneous operation of multiple ambulances has not caused 5G network congestion, and the service quality remains stable. This is mainly because the basic granularity of QoS in the current 4G network is an EPS bearer, which is essentially a logical aggregation of one or more business data flows. All data flows on the same bearer will receive the same QoS guarantee. There is a one-to-one relationship between EPS bearer and wireless bearer, and when there is a new QoS requirement, wireless as well as wired bearers need to be created simultaneously. The management of bearer control in the core network, on the wireless access network side, can only accept or reject bearer management requests from the core network. For 5G networks, the concept of QoS flows is introduced. Each QoS flow is marked with a QoS flow ID, which is unique during communication between a user terminal and a data network. There can be up to 64 QoS flows in a single session. Therefore, end-to-end user or business bandwidth and delay guarantees are provided through a unified QoS policy. In addition, 5G has two layers of QoS mapping; one for mapping IP flows to QoS flows and the other for mapping QoS flows to data wireless bearers. 5G QoS is controlled by the wireless side, which is more flexible and has a finer granularity, making it the key to solving latency and blocking issues in 5G.

5G medical devices are still in rapid development and production. If several devices need to simultaneously transmit data, the system’s uplink becomes a bottleneck. Therefore, we expect that new access technologies, such as carrier aggregation, will be used in the future to balance the downlink and uplink throughput.

### Comparison of 5G key indicators and 4G results

**Figure 9:**
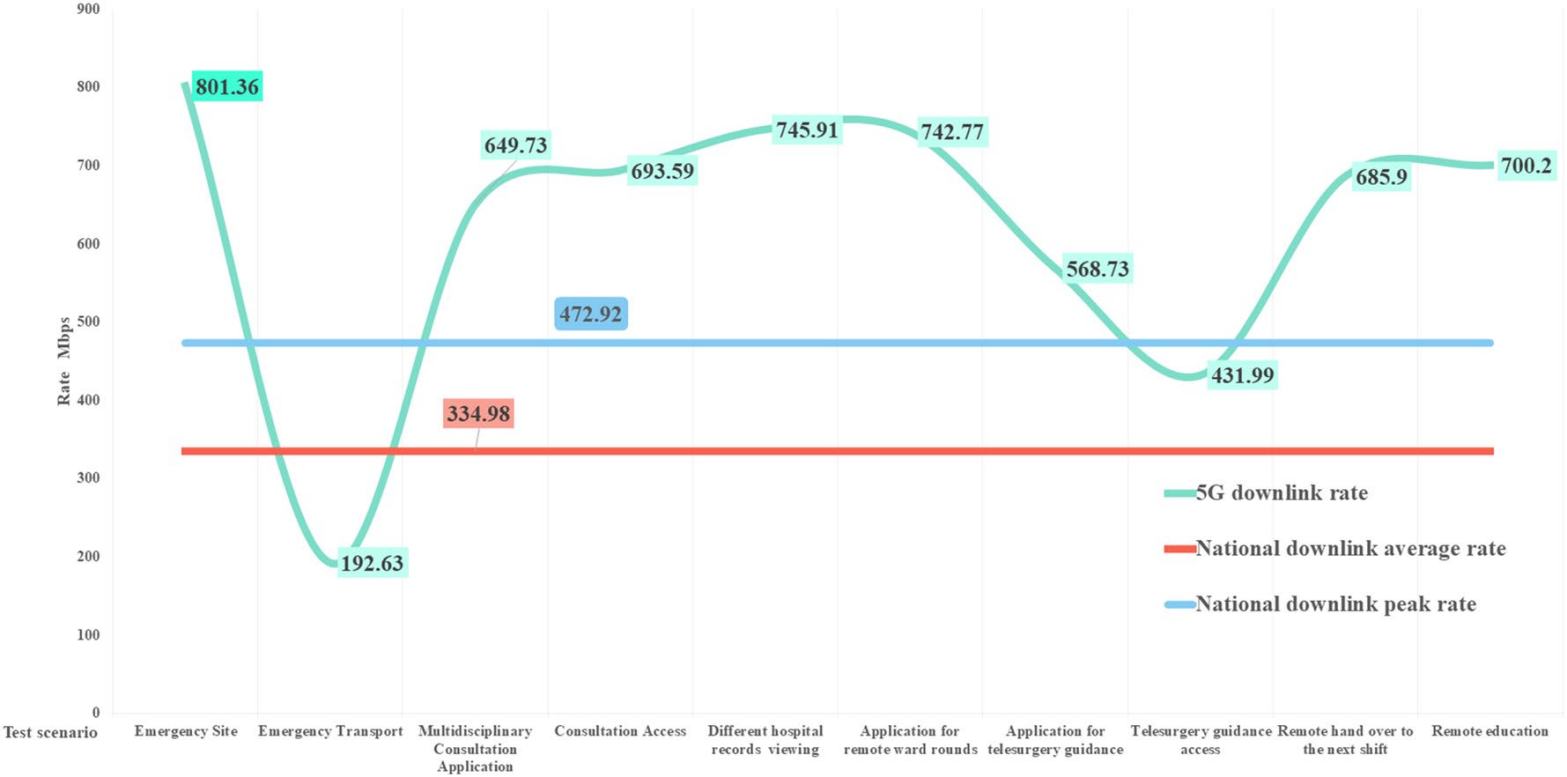
A comparison between the 5G downlink rate test value in the real scene and the national laboratory test average level.

**Figure 10:**
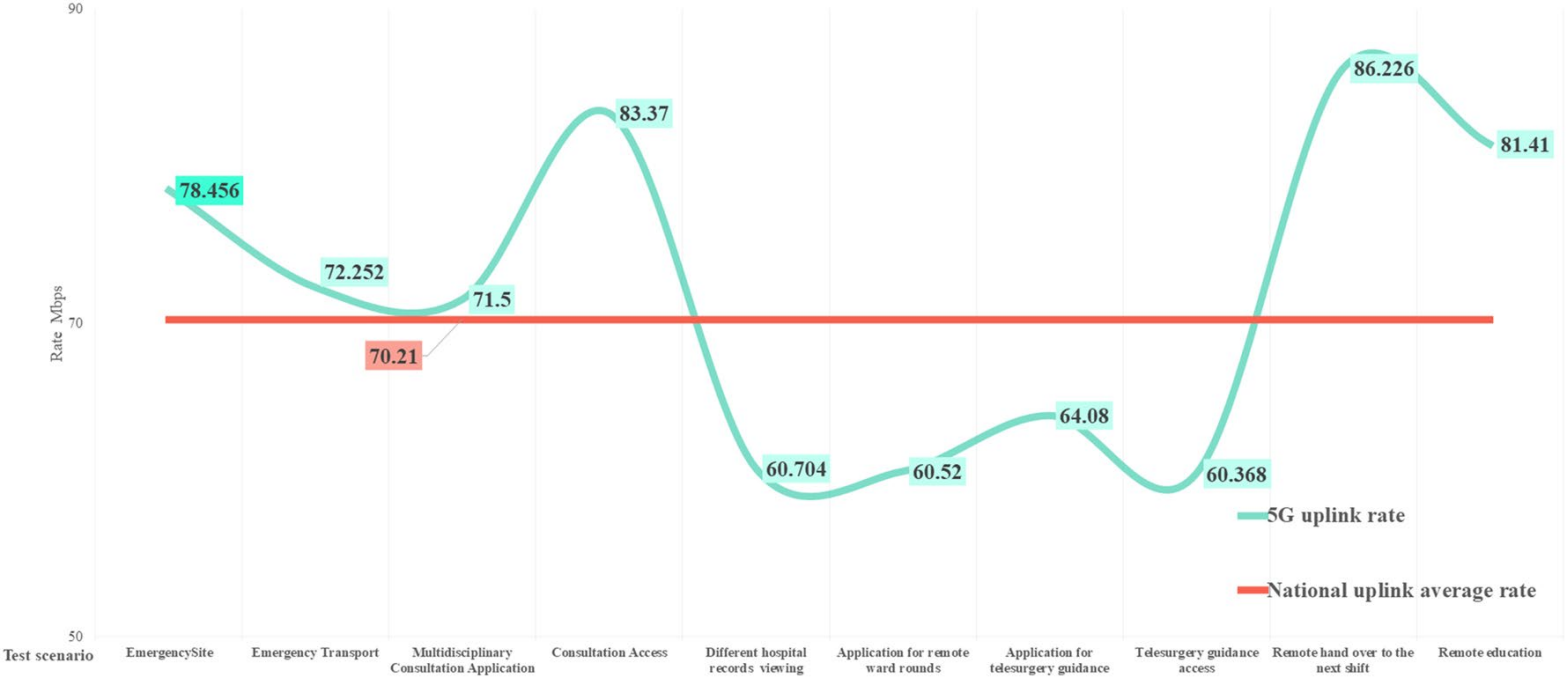
A comparison between the 5G uplink rate test value and the national laboratory test average level in real scenarios.

**Figure 11:**
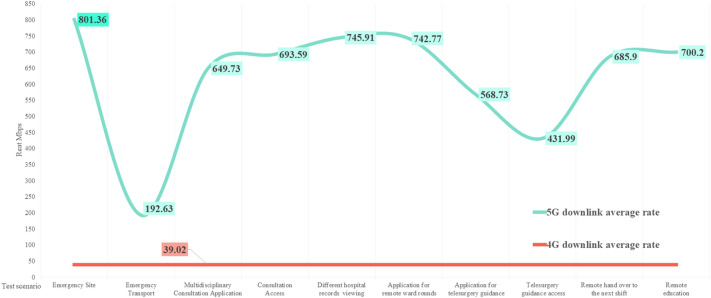
A comparison between the 5G downlink rate test value and the national 4G downlink rate average level in real scenarios.

**Figure 12:**
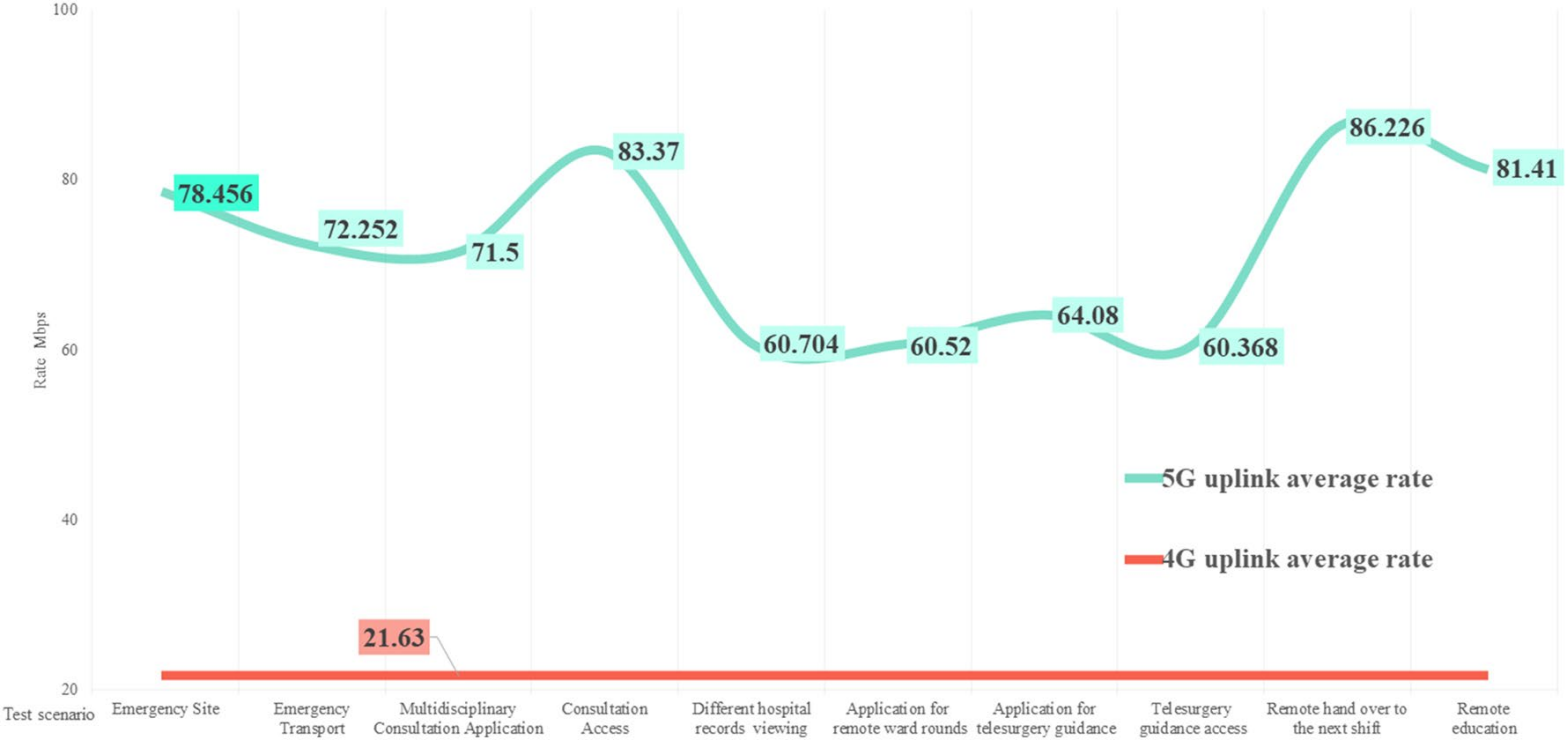
A comparison between the 5G uplink rate test value and the national 4G uplink rate average level in real scenarios.

According to the China mobile network quality monitoring report^43^ issued by the China Academy of Information and Communications in 2022, the national 5G network’s average downlink and uplink access rates are 334.98 and 70.21 Mbps, respectively; the downlink peak access rate is 472.92 Mbps. The comparison is shown in Figs. 9 and 10. Except for the impact of multiple mountain tunnels on the transportation process, the downlink peak in other scenarios is higher than the national average. The uplink rate is identical to the National Laboratory measurement results. The average downlink access rate and uplink access rate of the national 4G network are 39.02 and 21.63 Mbps, respectively, as shown in Figs. 11 and 12. The downlink access rate of 5G and 4G can be increased by up to 20 times.

### Fallback plan for emergency areas not covered by 5G during transportation

**Figure 13:**
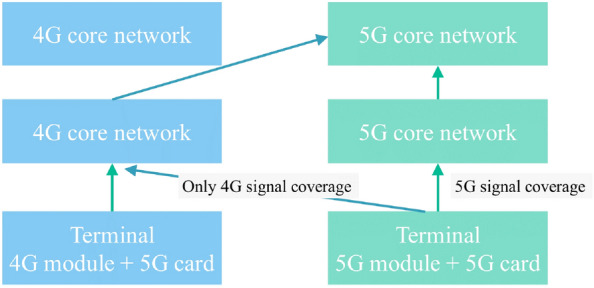
A 5G network fallback 4G network architecture.

In the case of mountain tunnels with low network frequency during emergency transport, the terminal shall have preferential access from the 5G base station. However, when the 5G signal is weak and only the 4G signal is covered, the terminal shall access the 4G base station and return to the 5G core network. This is shown in Fig. 13.

### Optimization of pre-diagnosis through AR technology

AR smart glasses can instantaneously identify patients' identities through face recognition, and merge them with their medical records. This can enable pre-diagnosis before the actual consultation, thus reducing the workload of doctors and freeing up more medical resources for patient treatment. To ensure accurate identification through AR smart glasses, the following methods are used: (1) High-quality image capture of the face: Currently, the face images used are pre-captured and are high-quality recent photographs, which substantially improves the accuracy of identification. (2) Double-checking of patient identity: The patient's identity is always double-checked, either through verbal confirmation by the doctor or by comparing it with their identification documents. (3) Continuous optimization of face recognition algorithms: The system supports the marking of identification errors as training data to optimize the recognition algorithm and improve accuracy. To prevent data contamination caused by identification errors, medical records are merged only after a manual review has verified the correctness of patient identity. Emergency medical records and historical medical records are stored in a specialized database to prevent contamination of historical data. In the future, AR smart glasses will continue to explore the combination of evolutionary computing and patient medical history data to automatically analyze patient conditions and assist doctors in making faster decisions by adapting pre-diagnostic results based on data features.

### Ethics approval and consent to participate

The study was approved by the Medical Science Research Ethics Committee of Peking University Third Hospital (serial number: IRB00006761-M2021476). Informed consent from the patients was exempt due to the study’s retrospective nature. All methods were performed under the relevant guidelines and regulations. The informed consent was waived by Peking University Third Hospital.

## Conclusions

Based on regional rescue information sharing, 5G synchronous transmission, and BeiDou Positioning Technology, we established a 5G transmission network-based emergency medical information sharing platform. It provided rapid identification, intelligent diagnosis and treatment, and remote consultation for the injured. Moreover, it realized the safe transmission of collected information, transmission, processing, storage, and utilization of the whole process, in addition to data sharing across institutions. This study’s proposed scheme supported the emergency medical support work in the multi-competition areas of the Winter Olympic Games and helped improve the treatment rate of the injured. According to the real scene’s test results, the 5G network satisfies the requirements of the pre-hospital emergency system application for the bandwidth, delay, and other network performance elements. Its main advantages are embodied in many aspects. First, based on BeiDou, the geographic location was accurately and promptly acquired from the real-time positioned vehicle, implying that it could automatically provide accurate and real-time spatial locations. According to the command center's dispatching information, traffic congestion was avoided and the dispatching time was significantly shortened. Second, AR smart glasses were used at the accident scene. The doctors of the destination hospital could grasp the patient's status and the real-time accident scene via the panoramic view. Third, the proposed system could provide real-time synchronization of vital signal data. If the patient has electrocardiogram monitoring changes, the expert group of the receiving hospital can grasp the real time-changes in the patient's condition, intervene, and guide the treatment in advance, improving the first aid quality. Finally, the scheme’s performance was verified via the 5G real application scenario test.

## Supplementary Information

Supplementary Information.

## Data Availability

Data supporting this study’s findings are available from the corresponding author upon reasonable request.
