# Supplementary material for: Method and application of information sharing throughout the emergency rescue process based on 5G and AR wearable devices
Source: Sci Rep. 2023 Apr 18;13:6353. doi: 10.1038/s41598-023-33610-4 (PMC10113192; doi:10.1038/s41598-023-33610-4)
Supplement: Supplementary file 1 — Supplementary Information. [file 41598_2023_33610_MOESM1_ESM.docx]

**Appendix 1**

| Test Scenario Indicators | | | |
| --- | --- | --- | --- |
| 1. Intra-Hospital Test Scenario | | | |
| Number | Indicator name | Unit | Indicator meaning |
| 1.1 | Signal coverage | % | Tests the system deployment site’s 5G network coverage. This indicator is used to verify whether the test point has completely covered by the 5G network signal. |
| 1.2 | SA Connection Success Rate | % | Tests the proportion of the terminal that can successfully access the 5G network. This indicator is used to verify the network’s stability. |
| 1.3 | SA dropout rate | % | The proportion of the test terminal dropping from the 5G network. This indicator is used to verify the network’s stability. |
| 1.4 | SA switch success rate | % | Tests the proportion of successful channel switching of the terminal. This indicator is used to reflect the channel handover of the 5G network. |
| 1.5 | 5G length of stay ratio | % | Tests the dwell time ratio of the terminal after accessing the 5G network. This indicator is used to verify the network’s stability. |
| 1.6 | SS RSRP (reference signal receiving power) | dB | The received power of synchronous signal reference is a key parameter that measures the wireless signal’s strength. |
| 1.7 | SS SINR (signal to interference plus noise ratio) | dB | The ratio of the strength of the received useful signal to the that of the received interference signal. |
| 1.8 | 5G downlink average rate | Mbps | The average downlink throughput is used to reflect the service quality of downlink data. |
| 1.9 | 5G uplink average rate | Mbps | The average uplink throughput is used to reflect the service quality of uplink data. |
| 1.10 | Packet loss | % | The proportion of the number of lost data packets in the sent data group in the data service test. This indicator is used to reflect the stability of data transmission. |
| 1.11 | Error rate | % | The initial block error rate of the data service. This indicator is used to reflect the accuracy of data transmission. |
| 2. Voice Service | | | |
| 2.1 | EPS fallback on rate | % | The proportion of the successful times of EPS fallback on the calling side to the total attempts. This indicator is used to reflect the success rate of voice service connection. |
| 2.2 | EPS fallback call delay | sec | This indicator is used to reflect the response speed perceived by the voice service user. |
| 2.3 | After the EPS fallback hangs up, the fast return delay is less than 3S | % | This indicator is used to reflect the speed at which the NR network switches from the 4G service state to the 5G idle one. |
| 2.4 | Error rate | % | The uplink channel block bit error rate of the voice service is used to reflect the accuracy of voice signal transmission. |
| 2.5 | RTP packet loss | % | The RTP packet loss rate is used to reflect the stability of voice call transmission. |
| 2.6 | RTP end-to-end delay | sec | The time difference between two RTP data packets is used to reflect the RTP network delay. |
| 3. Streaming Media Service | | | |
| 3.1 | Drop rate | % | The drop rate during video playback. This indicator is used to reflect the network’s stability. |
| 3.2 | Playback start failure rate | % | The failure rate of video playback start. This indicator is used to reflect the business connection’s success rate. |
| 3.3 | Play freeze rate | % | The proportion of video freezes when playing. This indicator is used to reflect the smoothness of the streaming media service. |
| 3.4 | Play start delay | sec | The loading delay at the beginning of video playback. This indicator is used to reflect the response speed of the streaming media service. |
| 3.5 | RTT delay | sec | The delay from the network side initiating the service to receiving the successful response from the terminal side. This indicator is used to reflect the response speed of the network and the terminal. |
| 3.6 | Video Play vMOS | -- | This indicator comprehensively considers the quality of video source, the initial delay in the playing process, and the percentage of stuck time, in addition to scoring the overall video experience. |
